# Supplementary material for: Identification of Long Non-Coding RNA-Associated Competing Endogenous RNA Network in the Differentiation of Chicken Preadipocytes
Source: Genes (Basel). 2019 Oct 12;10(10):795. doi: 10.3390/genes10100795 (PMC6826404; doi:10.3390/genes10100795)
Supplement: Supplementary file 1 [file genes-10-00795-s001.zip › Data S1. The arguments and the thresholds used to identify lncRNAs and miRNAs.docx]

The arguments and the thresholds used to identify lncRNAs

*TopHat2*

1) Maximum read mismatch is 2

2) The distance between mate-pair reads is 50 bp

3) The error of distance between mate-pair reads is ±80 bp

*CPC*

arguments: default

Thresholds: Transcript that with score greater than 0 is defined as mRNA, and transcript that with score smaller than 0 is defined as lncRNA.

*CNCI*

arguments: default

Thresholds: Transcript that with score greater than 0 is defined as mRNA, and transcript that with score smaller than 0 is defined as lncRNA.

*Blastall 2.2.25*

arguments: default

Thresholds: Reads that have identity more than 97% to rRNA, scRNA, snoRNA, snRNA, and tRNA were removed.

*bowtie(version 1.1.2)*

arguments: -v 0 --best --strata -a

v represents the naximum read mismatch.

*MIREAP_v0.2*

-A 18 Minimal miRNA sequence length (nt)

-B 26 Maximal miRNA sequence length (nt)

-a 20 Minimal miRNA reference sequence length (nt)

-b 24 Maximal miRNA reference sequence length (nt)

-c 3 Minimal depth of Drosha/Dicer cutting site

-u 20 Maximal copy number of miRNAs on reference

-e -18 Maximal free energy allowed for a miRNA precursor (kcal/mol)

-d 35 Maximal space between miRNA and miRNA* (nt)

-p 14 Minimal space between miRNA and miRNA* (nt)

-v 4 Maximal bulge of miRNA and miRNA* (nt)

-s 5 Maximal asymmetry of miRNA/miRNA* duplex (nt)

-f 10 Flank sequence length of miRNA precursor (nt)
