# Supplementary material for: Identification of Long Non-Coding RNA-Associated Competing Endogenous RNA Network in the Differentiation of Chicken Preadipocytes
Source: Genes (Basel). 2019 Oct 12;10(10):795. doi: 10.3390/genes10100795 (PMC6826404; doi:10.3390/genes10100795)
Supplement: Supplementary file 1 [file genes-10-00795-s001.zip › Data S2.Normal distribution test of RT-qPCR data of PPARG and FABP4.docx]

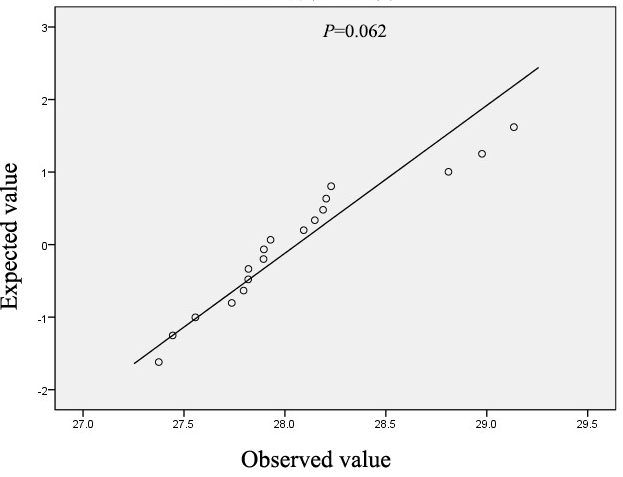
**Normal distribution test results**

Figure 1. Normal distributiom test of the RT-qPCR data of PPARG gene. The Q-Q plot and Kolmogorov-Smirnov test showed that the RT-qPCR data of PPARG gene conformed to normal distribution (*P*=0.062>0.05).


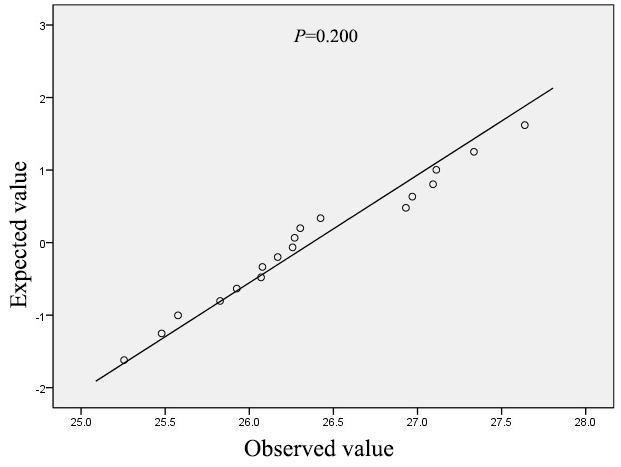


Figure 2. Normal distributiom test of the RT-qPCR data of FABP4 gene. The Q-Q plot and Kolmogorov-Smirnov test showed that the RT-qPCR data of FABP4 gene conformed to normal distribution (*P*=0.200>0.05).
